# Charting light harvesting in purple bacteria in vivo

**DOI:** 10.1073/pnas.2537487123

**Published:** 2026-06-29

**Authors:** Romain Rouxel, Julian Lüttig, Michael R. Jones, Donatas Zigmantas

**Affiliations:** ^a^ Division of Chemical Physics, Department of Chemistry, Lund University, Lund SE-221 00, Sweden; ^b^ Department of Physics, University of Ottawa, Ottawa, ON K1N 6N5, Canada; ^c^ School of Biochemistry, Biomedical Sciences Building, University of Bristol, Bristol BS8 1TD, United Kingdom

**Keywords:** multidimensional spectroscopy, light harvesting, energy transfer, photosynthesis, purple bacteria

## Abstract

Understanding the remarkable quantum efficiency of solar energy collection in phototrophic bacteria requires a clear description of the functional connectivity between the individual complexes comprising the photosynthetic apparatus. While significant progress has been made since the 1980s in understanding energy transfer dynamics within isolated complexes, information about intercomplex processes in intact cells is still sparse. One reason is the great complexity of intact systems that leads to highly congested spectra, especially at room temperature. Two-dimensional electronic spectroscopy (2DES) is a method that is well suited to discriminating between different subsystems and disentangling their responses, since the excitation energy is resolved along the “second dimension” of the spectra. However, 2DES on intact photosynthetic systems such as whole bacterial cells is challenging due to the high scattering properties of such samples. With a 2DES setup designed to reduce scattering, we were able to overcome these limitations. Here, we present physiological temperature measurements on intact cells of the purple photosynthetic bacterium *Rhodobacter sphaeroides*, which enabled us to fully map the energy transfer processes in an intact photosynthetic unit with femtosecond resolution, from the initial light-harvesting steps in the antenna complexes LH2 and LH1 through to arrival of the excitation to the reaction center. In particular, we observe the appearance of a clear signature of charge separation in the reaction center as a strong electrochromic band-shift signal. Through a global analysis approach, we extract the effective time constants for all these processes and expand the existing picture of how bacterial light harvesting works in vivo.

## Significance

Two-dimensional electronic spectroscopy emerged as the method of choice to unravel molecular couplings and energy transfer pathways in photosynthetic systems. Experiments on individual photosynthetic complexes have been very successful, but provided no information on the complete network of energy transfer pathways in intact photosynthetic organisms, containing multiple protein complexes. We overcame the challenge of measuring highly scattering cellular structures and mapped the complete excitation dynamics in the living cells of purple bacteria, starting from light absorption in the antennae complexes to charge separation in the reaction centers. Our results provide a full picture of primary photosynthetic processes in vivo and thus reveal the principles of highly efficient energy collection and conversion, which can inform the design of artificial energy conversion systems.

The photosynthetic apparatus of purple bacteria has received in-depth attention from spectroscopists over the past four to five decades, most intensively in the 1990s with the extensive use of ultrafast time-resolved spectroscopy techniques (1). The structures of individual components of the photosynthetic apparatus have been resolved by X-ray crystallography for various species of purple bacteria (2–8) and were found to be relatively simple compared to those of plants, which is why purple bacteria have often been chosen as a model system for photosynthetic research. In *Rhodobacter* (*R.*) *sphaeroides*, the photosystem consists of multiple pigment–protein complexes assembled within an extensive membrane system comprising connected spherical intracytoplasmic vesicles. Light is collected by two types of light-harvesting complexes called LH1 and LH2. LH2 contains two rings of 9 and 18 bacteriochlorophyll *a* (BChl) pigments, referred to respectively as B800 and B850 from their peak absorption wavelengths (2, 4). Additionally, carotenoids are incorporated into these systems, mainly playing a photoprotective role (9). The exact arrangement of B800 and B850 pigments is maintained by a protein scaffold spanning the membrane (3). LH1 has an S-shaped overall architecture and contains 56 B875 BChl pigments encircling two reaction centers (RCs) (6, 7). The RCs collect excitation energy from the surrounding LH1 complexes and perform charge separation. Excitation transfer dynamics within each complex have been thoroughly studied using time-resolved spectroscopy of isolated complexes in solution (1, 10, 11). However, learning about the functional connectivity between all the different parts, and thus obtaining a holistic view of the energy transfer pathways, requires studies of the whole photosynthetic unit.

The possibility to isolate intracytoplasmic membrane vesicles in solution by rupturing cells using a French press sparked a large number of time-resolved studies using such membranes (10). Although these studies were able to track energy transfer through the light-harvesting antenna, one must bear in mind that the membranes are in general not intact and may reorganize once isolated from the cells (12, 13), thus motivating the study of intact cells. Spectroscopic measurements of whole cells are notoriously difficult due to their size (a few micrometers) resulting in intense light scattering, in addition to their spectral complexity leading to congested spectra. The latter point is particularly true at room temperature due to spectral broadening. Despite this, investigation at room temperature is crucial to get an accurate picture of physiologically relevant dynamics, as use of cryogenic temperatures can, for instance, shift energy levels, stop diffusion processes, or block thermally allowed uphill transitions. Using time-resolved fluorescence spectroscopy, several studies were successfully conducted at physiological temperature on cells of various species of phototrophic bacteria (14–16), including *R. sphaeroides* (12), providing insights into the rate and efficiency of the overall energy trapping in whole cells. However, gaining precise information about the energy transfer pathways and timescales requires absorption-based techniques, which are more affected by sample scattering. Such information was recently obtained for several bacterial species using two-dimensional electronic spectroscopy (2DES) in setups employing various scattering reduction strategies (17–19). Compared to transient absorption spectroscopy, 2DES provides a spectral map not only on the detection energy axis but also on the excitation energy axis, which substantially facilitates the interpretation of congested signals, with excellent time and energy resolution. In their 2DES study from 2017, Dahlberg et al. (20) proposed estimates for most of the energy transfer rates in whole cells of *R. sphaeroides* and found them to be in agreement with the existing literature on isolated complexes and membranes. However, as in all previous studies on intact cells or on membranes, no signature of charge separation in the RC was reported. Thus, arrival of the energy to the RCs has never been detected for any bacterial species in experimental systems that preserve the photosynthetic unit in its native form.

Here we present room temperature 2DES measurements of energy transfer and trapping in whole cells of *R. sphaeroides*. Using a dedicated 2D setup that employs lock-in detection and sample motion, we obtain 2D spectra with considerably suppressed scattering and at low excitation intensities, and consequently low exciton–exciton annihilation, while maintaining a high signal-to-noise ratio. The data, scrutinized using global analysis, show the distinctive signatures of all the main intra- and intercomplex energy transfer processes, as well as charge separation within the RC, and allows extraction of the associated effective timescales as they occur in living bacteria. We discuss the significance of our conclusions in relation to what is currently known or assumed about light harvesting in these bacteria.

## Experimental Results.

**Fig. 1.**
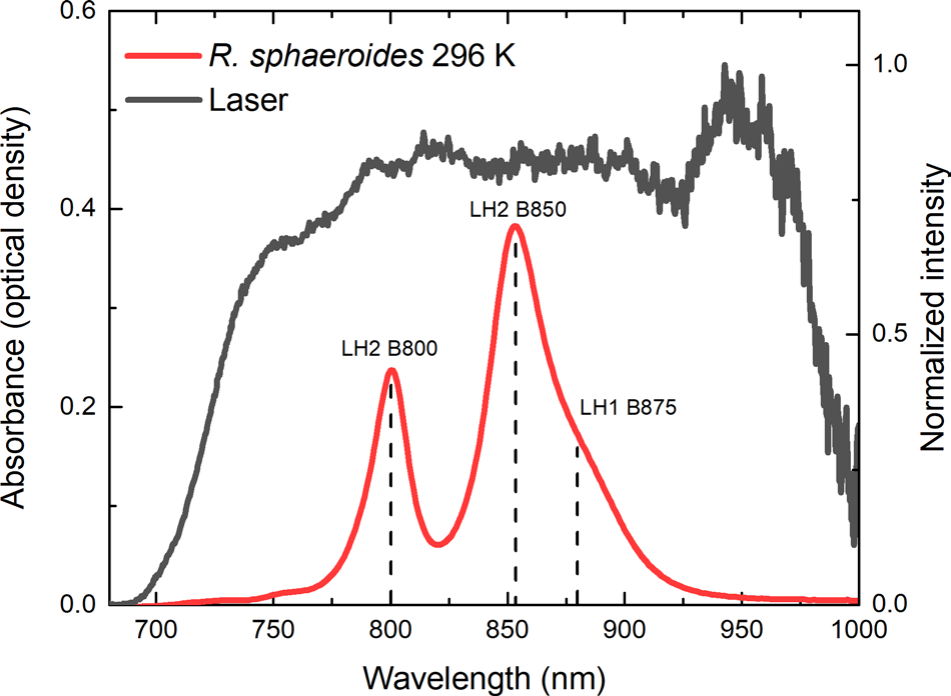
296 K absorption spectrum of photosynthetically grown *R. sphaeroides* cells (red line). The laser spectrum (beams 1 to 4) is shown in gray. The dashed lines indicate the centers of absorption bands found in the LH1 and LH2 complexes, corresponding to the main electronic transitions.

The sample absorbance spectrum before the 2DES measurement is presented in Fig. 1. The three main absorption peaks from the antenna complexes LH2 (B800 and B850) and LH1 (B875) are visible. The spectrum recorded after the measurements, shown in the *SI Appendix*, Fig. S1, indicated a notable decay of the B800 absorption peak while the B850 and B875 bands were unchanged. However, as discussed in the *SI Appendix*, we find that the progressive decay of B800 has a negligible influence on the dynamics of the system recovered by global analysis. The laser spectrum used in the 2DES measurements (Fig. 1, gray curve) covers all three transitions.

**Fig. 2.**
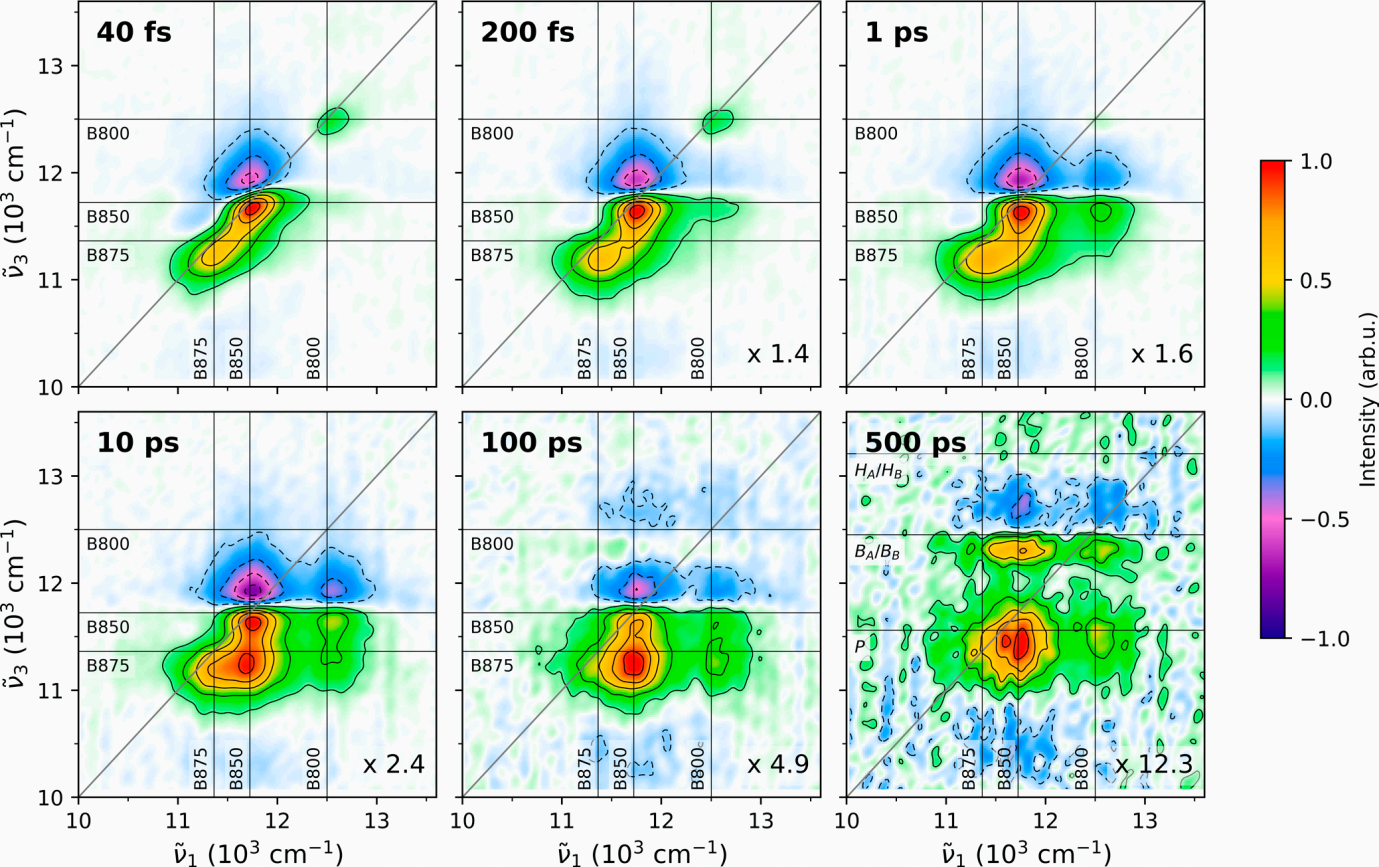
Real (absorptive) part of the total (sum of the rephasing and nonrephasing parts) 2DES signal of *R. sphaeroides* cells at 296 K, shown at several population times $t_{2}$ as indicated on the *Top Left* of each graph. The color scale of each map is normalized to its maximal value. The multiplication factor to attain the maximum level of the 40 fs map is given on the *Bottom Right*. Contour lines are plotted between −0.9 and 0.9 with 0.2 step. Horizontal and vertical lines indicate the main electronic transitions found in LH1 and LH2 (11,360 cm^−1^ for the B875 band of LH1, and respectively 11,720 and 12,500 cm^−1^ for bands B850 and B800 of LH2, corresponding to the peaks in the linear absorption spectrum). At $t_{2}=$ 500 ps, horizontal lines correspond to the main RC transitions (11,560, 12,450, 13,210 cm^−1^, obtained from ref. 25).

Fig. 2 shows the absorptive 2D spectra for a selection of population times. By convention, ground-state bleach (GSB) and stimulated emission (SE) appear as positive signals, while excited-state absorption (ESA) gives rise to negative signals. Population times shorter than 40 fs are not considered in our dataset due to artifacts originating from the ambiguous time ordering in the pulse overlap region and nonresonant signals (20–24). At *t*_2_ = 40 fs (Fig. 2), the 2D spectrum indicates direct excitation of the antenna complexes. The positive diagonal signals at 11,360 cm^−1^, 11,720 cm^−1^, and 12,500 cm^−1^ correspond to GSB and SE of the B875 excitonic state of LH1, and the B850 and B800 states of LH2, respectively. Above-diagonal negative peaks at (${\tilde{\nu }}_{1}$, ${\tilde{\nu }}_{3}$) = (11,720 cm^−1^, 11,950 cm^−1^) and (11,200 cm^−1^, 11,600 cm^−1^) indicate strong ESA from the B850 state and much weaker ESA from LH1.

Between 200 fs and 1 ps, excitation energy transfer is observed from the B800 ring to the B850 ring. It manifests as a decay of the B800 diagonal peak at (12,500 cm^−1^, 12,500 cm^−1^) concomitant with the appearance of two below-diagonal positive (12,500 cm^−1^, 11,650 cm^−1^) and negative (12,500 cm^−1^, 11,960 cm^−1^) peaks. These peaks respectively correspond to (GSB, SE) and ESA from B850. In the next few tens of picoseconds, further energy transfer occurs from the B850 ring of LH2 to LH1, seen in the $t_{2}$ = 10 ps data as the emergence of positive off-diagonal peaks at (11,720 cm^−1^, 11,360 cm^−1^) and (12,500 cm^−1^, 11,360 cm^−1^). Finally, energy transfer toward the RC takes place, followed by the charge separation processes. Because the absorption bands of the RC largely overlap with the three bands of LH1 and LH2, the intermediate 2D spectra (e.g., $t_{2}$ = 100 ps) are less straightforward to analyze. However, the spectrum measured at $t_{2}$ = 500 ps (Fig. 2) clearly shows a fully realized charge-separated state in the RC, its 2D spectral shape being very similar to previously measured 2D spectra of isolated RCs (26, 27). This final state is clearly independent of the initially excited transition, leading to almost identical spectral profiles along the detection axis. According to the current picture of charge separation, excitation transfer from a LH1 complex to the special pair of BChls in the RC, referred to as P, quickly leads to the oxidation of P to P^+^, as an electron from P is sequentially transferred to accessory BChl B_A_, to bacteriopheophytin H_A_ and finally to quinone Q_A_ (26, 27). In the 2D spectrum at *t*_2_ = 500 ps, the wide positive peaks around ${\tilde{\nu }}_{3}$ = 11,500 cm^−1^ correspond to GSB from the formerly excited P* state, following charge separation (26, 27). These signals are very broad due to interaction of the special pair with the surrounding polar environment. The pairs of close positive and negative peaks around 12,300 cm^−1^ and 12,700 cm^−1^ detection wavenumbers are clear signatures from the electrochromic shifts of the B_A_ and B_B_ bands induced by the local electric field in the charge-separated P^+^Q_A_^−^ state. As for the negative region around ${\tilde{\nu }}_{3}$ = 10,500 cm^−1^, it could originate from a slight phasing error, as it is present at all population times.

## Data Analysis.

To obtain further insight into the energy transfer processes, and in particular to extract their characteristic timescales, we performed global analysis of the 2D spectra (28). This method consists of fitting the entire dataset with a sum of *N* components comprised of a purely temporal part $e^{-t_{2}/\tau_{j}}$ and a purely spectral part ${DAS}_{j}\left({\tilde{\nu }}_{1},{\tilde{\nu }}_{3}\right)$:

[1] $$\begin{array}{c} S\left({\tilde{\nu }}_{1},t_{2},{\tilde{\nu }}_{3}\right)=\sum_{j=1}^{N}{\mathrm{DAS}}_{j}\left({\tilde{\nu }}_{1},{\tilde{\nu }}_{3}\right)e^{-t_{2}/\tau_{j}}, \end{array}$$

where $S\left({\tilde{\nu }}_{1},t_{2},{\tilde{\nu }}_{3}\right)$ is the fit function, $\tau_{j}$ are time constants and ${DAS}_{j}\left({\tilde{\nu }}_{1},{\tilde{\nu }}_{3}\right)$ are the decay-associated spectra (DAS). Provided that the number *N* of exponentials has been chosen correctly and that all the processes involved are exponential and well-separated in time, the *N* components from the fit can each be attributed to a particular process, e.g., energy transfer or decay, taking place with a characteristic time $\tau_{j}$. Thus, the *N* corresponding DAS [the amplitude maps in the (${\tilde{\nu }}_{1}$, ${\tilde{\nu }}_{3}$) space] are used to characterize the energy transfer and relaxation processes. This method has been shown to provide fruitful information in complex systems (17, 26, 29–31), being especially useful when more than three time constants are involved in each (${\tilde{\nu }}_{1}$, ${\tilde{\nu }}_{3}$) “point” (kinetic trace over $t_{2}$) of the 2D spectra. In such a scenario, fitting of single kinetic traces is not reliable and usually fails to provide time constants that are the same on a larger region of the map, as was found in the present case when attempting to use this method. By fitting all of the data simultaneously, global analysis correlates the kinetics at the different points of the map and is therefore able to extract time constants more reliably. It should be noted, however, that these constants generally correspond to the effective average of many individual processes with a distribution of time constants, especially in systems as complex as the present one.

**Fig. 3.**
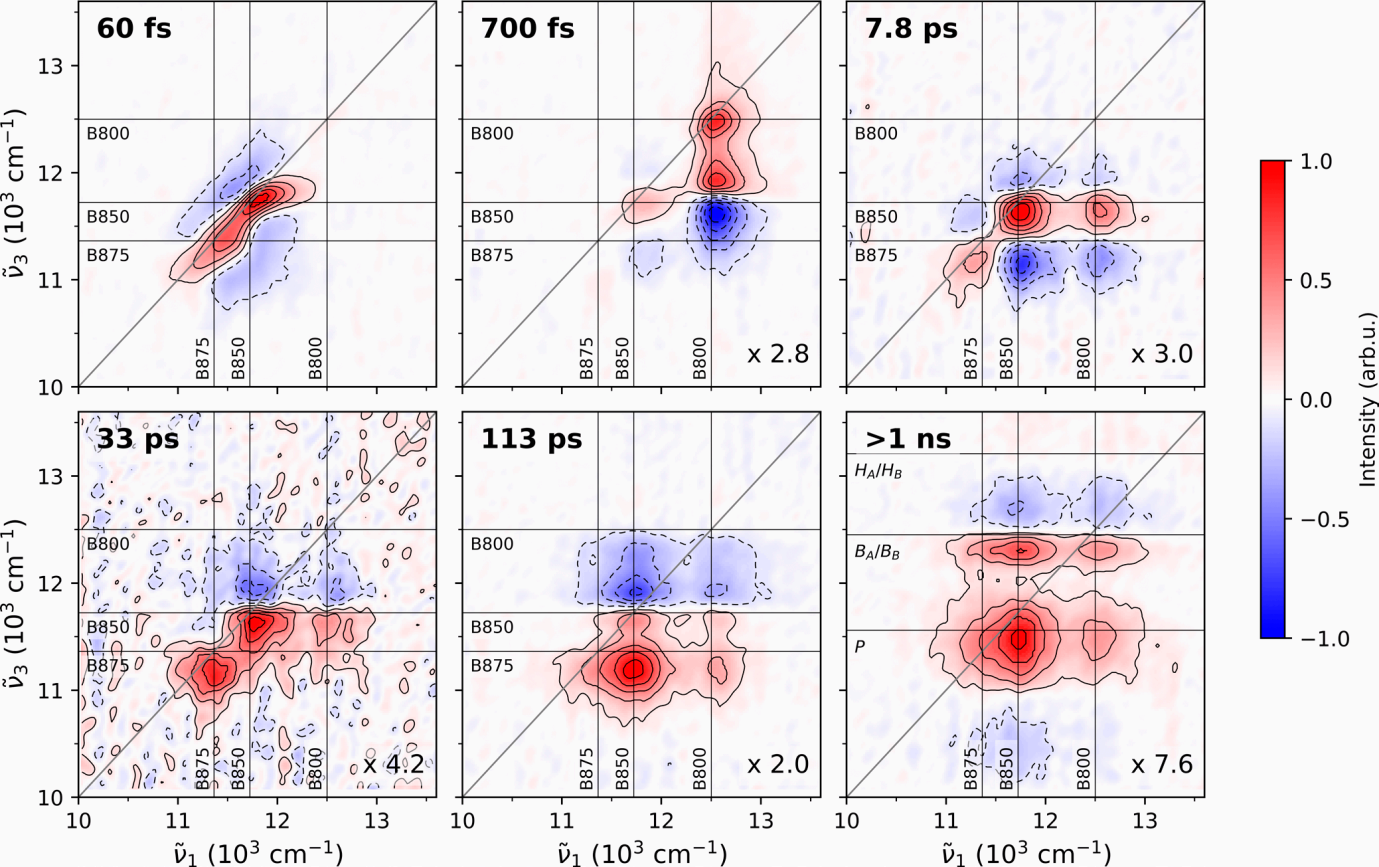
Decay-associated spectra (DAS) from the global analysis of the experimental data with six exponential decays. The time constants of each exponential are indicated on the *Top Left* of the maps. The color scale of each spectrum is normalized to its maximal value. The multiplication factor to attain the maximum level of the 60 fs component is given on the *Bottom Right*. Contour lines are plotted between −0.9 and 0.9 with a 0.2 step. Horizontal and vertical lines indicate the main electronic transitions of LH1 and LH2 for each component, except in the last component where horizontal lines correspond to RC transitions (Fig. 2).

The global analysis results are presented in Fig. 3. Six exponential components were necessary to account for the dynamics in the system, addition of a seventh component failing to lead to an emergence of an independent rate associated with an amplitude map with a different spectral shape. In contrast, the six isolated DAS present distinct signatures and can therefore be attributed to distinct physical processes. The shortest 60 fs component is attributed to spectral diffusion within the B850 and B875 rings of LH2 and LH1, respectively. The corresponding DAS shows positive peaks slightly below the diagonal at (11,820 cm^−1^, 11,750 cm^−1^) and (11,480 cm^−1^, 11,400 cm^−1^), as well as negative peaks above and below. Through comparison with the *t*_2_ = 40 fs slice of the 2DES signal (Fig. 2), the upper negative peaks and the positive diagonal peaks can be attributed to a decay of the ESA and (GSB, SE) signals, respectively, as the initially excited states relax to lower states of the same bands. Similarly, the lower negative peak is attributed to the corresponding increase of GSB and SE signals from these lower states. The effective time constant for this process, 60 fs, is consistent with previously reported measurements on isolated LH2 and LH1 complexes or on membranes (32, 33). The B850 and B875 excitonic states have been shown to be delocalized over ~4 BChl sites (10) and to relax within the same ring.

The 700 fs DAS shows the signature of the well-known energy transfer between the B800 and B850 rings of LH2. The positive diagonal peak at (12,500 cm^−1^, 12,470 cm^−1^) indicates a population decay of B800 while the below-diagonal positive (12,500 cm^−1^, 11,930 cm^−1^) and negative (12,500 cm^−1^, 11,620 cm^−1^) peaks correspond to the simultaneous increase of the ESA and (GSB, SE) of B850, respectively. The determined time constant is in excellent agreement with previous reports at room temperature in isolated LH2 complexes (10, 32, 34, 35). Additionally, the weak positive and negative peaks at (11,830 cm^−1^, 11,720 cm^−1^) and (11,830 cm^−1^, 11,230 cm^−1^) seem to marginally capture the end of B850 spectral relaxation, which has been found by several authors to have a longer 400 to 1,000 fs component in addition to the main 50 to 100 fs one (36, 37). The nonexponential decay of B850 relaxation has been reproduced previously with a model assuming incoherent hopping between dimeric sites (37).

The next process, described by an ~8 ps exponential decay, clearly corresponds to the energy equilibration between the LH2 and LH1 subunits. At 800 nm and 850 nm excitation wavelengths, population decay of the B850 state is observed, concomitant with a population increase of B875, seen as an increase (negative peaks) of the GSB and SE signals of B875 at (11,770 cm^−1^, 11,160 cm^−1^) and (12,500 cm^−1^, 11,160 cm^−1^). At 875 nm excitation, the reverse process is also observed, but with much lower amplitude, corresponding to the uphill transfer from the excited LH1 to the B850 ring of a nearby LH2. This transfer is weakly thermodynamically allowed, as its energy gap (~450 cm^−1^) is in the same range as thermal energy, k_B_T = 205 cm^−1^ at 296 K. Because an excitation can be transferred through several LH2 complexes before reaching an LH1 antenna, the dynamics for the overall transfer of energy to LH1 are not strictly exponential. In previous experimental studies, the energy transfer via several LH2 complexes has been described either by a single exponential (38, 39), or two exponentials (40, 41), with a short (3 to 10 ps) and/or a long (25 to 50 ps) component being reported, either separately or conjointly. In our global analysis, the 8 ps time constant captures very well the dominating timescale of LH2 to LH1 energy transfer and constitutes a reliable estimate for this transfer time in our system. This parameter could, however, depend on the extent and structure of the antenna pool, which are known to vary in different bacteria species and according to the growth conditions (42–44).

The ~30 ps component of the global analysis has a very similar appearance to the 1 ps 2D spectrum, where the directly excited LH1 and LH2 complexes are found in their fully relaxed state. In the DAS, such a picture must be interpreted as a population decay of these bands, while no other feature in this DAS indicates a corresponding rising signal. This decay is unlikely to be caused by fluorescence of the LH1 and LH2 complexes since the fluorescence lifetimes of LH1 and LH2 were measured to be ~1,000 ps in mutant membranes containing only LH1 or LH2 (45, 46). Thus, we attribute this decay to exciton–exciton annihilation. This attribution was strongly supported by an additional 2DES measurement with a fourfold higher pulse energy (2 nJ per pulse, corresponding to a fluence 4.4 μJ cm^−2^ per pulse). In this additional measurement, a similar component was found in the global analysis, though featuring a much higher relative amplitude and a shorter timescale (see *SI Appendix*, Fig. S5 for details). The presence of annihilation despite the low excitation fluence used in our main measurement (1.1 μJ cm^−2^ per pulse), is in contrast with a previous 2DES study of *R. sphaeroides* cells, where the measurements were deemed annihilation-free for fluences of up to 5.6 μJ cm^−2^ with a similar excitation spectrum (19). However, at a higher power (17.6 μJ cm^−2^), the authors of that previous study noted a clear signature of annihilation in their data, with the appearance of a decay component on a timescale compatible with our observation (~40 ps), in both LH1 and LH2 bands (19). We interpret the fact that we could detect annihilation at a much lower excitation density as being a consequence of the high sensitivity of our measurement system combined with long-time averaging, as well as our robust analysis method. Despite the relatively low amplitude of this annihilation component (4.2 times lower than the 60 fs spectral diffusion component), we found that it has a notable influence on the closest time constants in the global analysis, as further discussed in the *SI Appendix*, Figs. S3 and S4. Our method thus leads to better estimates for the time constants relevant to energy transfer. However, one should note that the ~30 ps exponential fit only captures the effective dynamics of exciton–exciton annihilation which, like LH2-LH1 transfer, is a diffusion-related process.

The ~110 ps decay is attributed to the energy transfer between LH1 and the special pair P in the RC and to the subsequent charge separation. Since charge separation is much faster [~2 to 3 ps (26, 47)], the two events cannot be disentangled, and the extracted time constant corresponds to the limiting step of the LH1 → P energy transfer. The corresponding DAS has a rather complex spectral shape whose vertical cross-section is largely independent from the initially excited band. It is better understood by comparing the initial and final stages of the process, seen in the 2D spectra at 10 ps and 500 ps, respectively (Fig. 2). The “uphill” transfer between the spectrally close LH1 B875 and RC P870 bands are expected to appear as a positive and negative double peak around ${\tilde{\nu }}_{3}$ = 11,500 cm^−1^, characteristic of the blueshift. The positive part is indeed visible around ${\tilde{\nu }}_{3}$ = 11,200 cm^−1^, but the negative part is not visible in the DAS, possibly being hidden by the positive peaks with narrower bandwidth visible at (11,730 cm^−1^, 11,640 cm^−1^) and (12,500 cm^−1^, 11,640 cm^−1^). These signals, topped by negative peaks at (11,730 cm^−1^, 11,910 cm^−1^) and (12,500 cm^−1^, 11,910 cm^−1^), could correspond to residual exciton–exciton annihilation in the LH2 pool, which is known to contain zones largely disconnected from the LH1–RC complexes under certain growth conditions (48). The elongated negative peaks at (11,730 cm^−1^, 12,300 cm^−1^) and (12,500 cm^−1^, 12,300 cm^−1^) distinctly indicate the appearance of the electrochromic shift signals from B_A_ and B_B_ (26, 27) visible in the 2D spectrum at 500 ps.

The last component of the global analysis corresponds to the decay of the long-lived charge-separated state in the RC, and its shape is identical to the 2D spectrum at *t*_2_ = 500 ps (Fig. 2). The timescale of this process exceeds the measurement window and appears to be longer than a nanosecond. As currently understood, the RC reopening, which results in the recovery of neutral species in the RC, takes place on a timescale on the order of 1 ms and is highly dependent on oxidizing conditions, ionic strength, pH, and temperature (49, 50).

## Discussion

The measured transfer times of 60 fs for the internal relaxation within LH1 and the B850 ring of LH2, and of 700 fs for the energy transfer from B800 to B850 within LH2, are in good to excellent agreement with the literature. Our experiment also allows for a better characterization of the equilibration time between the LH2 and LH1 pools with a single time constant of ~8 ps, while available values in the literature are largely inconsistent. Note that we cannot infer the characteristic time for the energy transfer between adjacent LH2 complexes since this process is not connected to a change of the absorption spectrum and is therefore not directly visible in the isotropic 2DES experiments. In contrast with the other measured time constants, the 110 ps found for the LH1→RC energy transfer is longer than the 35 to 60 ps reported by other groups both at room temperature (12, 42, 51) and at cryogenic temperature (52–54), using various experimental strategies. The fundamental variance from our measurements is that all previous measurements only reported the population decay of the LH1 excitons and could not detect the corresponding RC signal increase. In contrast, we directly detect the RC signal rise that is correlated with the LH1 decay, which provides direct evidence of the LH1→RC transfer. However, the discrepancy between the time constants described above could also originate from the fact that a portion of the RCs in the present study are in a closed state due to the excitation conditions. Indeed, the exciton lifetime in the LH1 pool has been shown to increase when RCs are in a mixture of open and closed states, presumably because the excitons need to explore a larger portion of the membrane until finding an open RC, or eventually being quenched by a closed RC (12, 42, 45). When all RCs are closed, this lifetime reaches a maximum value of ~200 to 250 ps depending on the LH1/LH2 ratio (12, 42). In our experiment, both processes of energy transfer from LH1 to open and closed RCs could be mixed in the 110 ps DAS of Fig. 3, although their spectral signatures must be slightly distinct (as the RC charge-separation signal only appears when transfer takes place to a neutral P in the open RC). The 110 ps time constant could thus indicate that our system is in the mixed state of open and closed RCs observed in previous studies (12, 42, 45). We also note that residual annihilation in the LH1 and LH2 pools, whose weak signature was detected in the 110 ps DAS (Fig. 3), might also influence the extraction of this time constant.

Importantly, the absence of any signatures of long-lived excited states of LH1 and LH2 in our data, in particular, in the longest DAS component (Fig. 3) or in the 500 ps 2D spectrum (Fig. 2), points to an overall transfer efficiency close to 100% from the antenna complexes to the pool of RCs, whether open or closed.

It is difficult to estimate the ratio of open to closed RCs under our experimental conditions. To gain insight into this, we estimated the rate of RC excitation. First, the excitation probability of the different complexes was calculated through a measurement of the number of photons absorbed by the sample and using tabulated values for the extinction coefficients associated to each band of the spectrum (*SI Appendix*, Fig. S2 and Table S1). The calculation reached probabilities of direct excitation by the two pump pulses of ~2.5% per LH2 complex, 5.1% for the S-shaped LH1 complex, and 0.35% for the RC. The concentrations of LH1, LH2, and RCs were also estimated, corresponding to an LH2/RC ratio of ~3. Assuming a 95% quantum efficiency of trapping (55), this results in a ~10% probability of exciting the RCs per two pump pulses after complete energy transfer, which goes up to ~15% when adding the effect of the third (probe) pulse. Thus, each series of pulses should induce the closure of 15% of the RCs. Considering an RC turnover time of 1 ms (49), only partial reopening of the charge-separated RCs within the 55 μs interval between the pulses is expected, which should lead to rapid inactivation of all RCs. However, the presence of open RCs in our experimental conditions is indisputably evidenced by the signature signal of the charge separation state observed in our measurements. The presence of a renewed pool of open RCs throughout the measurement can be explained by a combination of different effects. These include the raster scanning motion of the sample, chopping of both excitation pulses at 200 and 400 Hz frequency, sample diffusion in the cuvette, as well as some RCs recovering from the previous excitation. This is aided by the sensitivity of the technique, enabling us to detect the weak signal from the population of RCs that are open during the energy transfer from the LH1 complexes.

It is also important to note that, while we estimate that a certain proportion of RCs are expected to be in a closed state, no clear background signal originating from this species is visible in our measurement. This is a consequence of our detection scheme, which employs lock-in detection using the modulation of the two excitation beams by optomechanical choppers. The lock-in detection suppresses the spectroscopic signatures of long-lived species such as triplets, thermal grating, or, in our case, closed RCs (for details see ref. 56). Importantly, the detection scheme only suppresses long-lived species without influencing fast relaxation processes like exciton transfer, which is crucial for the presented experiment. Without the suppression of long-lived species, we anticipate that signal from the closed reaction centers would substantially complicate the observation of energy transfer dynamics to open RCs.

In addition to partially closing RCs, excess excitation is also causing some exciton–exciton annihilation. Despite the low fluence used (1.1 μJ cm^−2^ per pulse), an annihilation signal is still present. In fact, annihilation seems very difficult to avoid in 2DES experiments on intact organisms, because the whole photosynthetic unit is very sensitive to annihilation effects due to the very large number of interconnected complexes and the efficient exciton diffusion between them. The estimated values of the excitation probabilities per complex provided above are compatible with the presence of a low level of exciton–exciton annihilation in the system. Double excitation of individual complexes is conversely very unlikely, as discussed in the *SI Appendix*. Thus, annihilation in our experiments is solely related to excitation diffusion between the complexes.

Additional measurements on the *R. sphaeroides* cells performed at 80 K are presented in the *SI Appendix*, Figs. S6–S8. Aside from the narrower absorption bandwidths resulting from the low temperature, the data, as well as the time constants and DAS derived from global analysis, are generally quite similar to those obtained from the room temperature measurements. However, RC charge-separation signals are strikingly absent from the spectra at a long population time (e.g., 500 ps) in the 80 K measurements. Instead, the excitation energy seems to be unable to reach the RCs and remains trapped in the antenna complexes until it decays via fluorescence [with a 220 ps time constant comparable with previously reported values for membranes with entirely closed RCs (57)]. The natural explanation for this is that all RCs are closed during the 80 K measurements. Importantly, the raster motion of the cuvette, contained in a cryostat, could not be implemented in this experiment. Whereas the cryostat can be moved in the direction perpendicular to the beams, such motion generates unmanageable amount of scattering, because of the multitude of microcracks in the low-temperature glass sample. Thus, a single spot of the sample was illuminated during the whole measurement lasting for several hours. Therefore, we explain the absence of the charge-separation signal in RCs by the lack of sample renewal during the 80 K measurement.

## Conclusion

**Fig. 4.**
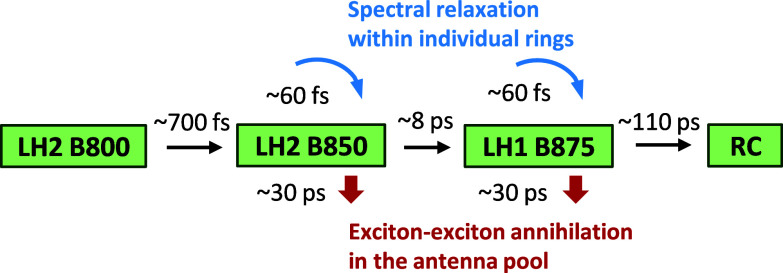
A summary of the timescales of energy transfer processes as determined from our experiments and analysis. The indicated time constants correspond to the effective (*i.e.*, multiprocess) energy transfer in the bacteria and not to a single-event process.

Our 2DES measurements highlight the full light-harvesting process in living cells of purple bacteria. Using a lock-in detection scheme and raster motion of the sample, we address several challenges associated with 2D spectroscopy of intact cells, such as intense scattering and signals from the accumulation of long-lived species. Furthermore, the achieved high signal-to-noise ratio allows for a substantial reduction of the pump intensity and consequently a sufficient decrease of exciton–exciton annihilation, as well as detection of weak RC band-shift signals. The effective time constants found by global analysis for the successive energy transfer steps generally agree with previous measurements performed either on membranes or on isolated complexes and are summarized in Fig. 4. After relaxation within the B850 and B875 BChl rings with a rate of 60 fs, energy transfer occurs between the B800 and B850 rings of LH2 with a 700 fs time constant. Energy equilibration between the LH2 and LH1 pools is described by an 8 ps component. The final transfer step to a mixture of open and closed RCs is characterized by an effective 110 ps time constant, and we are also able to observe clear signatures of the ensuing charge separation. Moreover, the absence of any signals from long-lived excited states of the antenna complexes indicates a near-100% transfer efficiency to the RCs. Despite the unusually low pulse energy used in our experiments, requiring averaging of the data, a low level of exciton–exciton annihilation was still present in the measurements, which is adverse for the precise characterization of energy transfer processes. Thus, it should be avoided as much as possible in any measurements of intact photosynthetic systems. Of note, a recently demonstrated nonlinear spectroscopy approach (58, 59) could provide an alternative to low-power measurements by enabling separation of the different orders of the nonlinear signals. In the future, it could be used to extract the annihilation-free dynamics as well as to study exciton diffusion within the pools of antenna complexes.

In summary, the findings reported in this paper highlight the capability of the scatter-resistant 2DES spectroscopy to track the energy transfer throughout the intact photosynthetic system at physiological conditions from initial light absorption by the antenna complexes to charge separation in the RC. The technique is not specific regarding the sample and could be applied to any photosynthetic organism in vivo.

## Materials and Methods

The 2DES setup used in this study has been described in a previous publication (56) and we briefly review the experiment here. A lab-made noncollinear optical parametric amplifier (NOPA) is pumped by a pulsed laser (Pharos, Light Conversion) and tuned to generate broadband near-IR pulses with a central wavelength of 850 nm and a bandwidth of 250 nm full width at half maximum (FWHM). The repetition rate is set to 18 kHz, chosen to avoid accumulation of signals from long-lived species and yet to reach sufficient signal-to-noise ratio. FWHM pulse duration is 14 fs at the sample position, determined by autocorrelation using a BBO crystal. The beam is split into four components whose polarizations are set to the magic angle configuration (54.7°,54.7°,0°,0°) to eliminate anisotropy effects. Beams 1 and 2 are referred to as the “pumps” and beam 3 as the “probe.” The delays between pulses from beam 2 and 3 (the population time, $t_{2}$) and between pulses from beam 1 and 2 (the coherence time, $t_{1}$) are controlled independently. The four beams are focused onto a ~200 μm FWHM-wide spot of the sample in a box-CARS configuration (60). The third-order nonlinear signal is coupled into a spectrometer equipped with a CCD camera. It copropagates with the attenuated beam 4, which plays the role of local oscillator and enables retrieving the amplitude and phase of the signal electric field through heterodyne detection. Intensity modulation of beams 1 and 2 using optomechanical choppers at respectively 200 Hz and 400 Hz allows for a drastic increase of the signal-to-noise ratio using multichannel lock-in detection. The 2DES signal as a function of the excitation and detection wavenumbers (${\tilde{\nu }}_{1}$, ${\tilde{\nu }}_{3}$) is obtained from the raw ($t_{1}$, ${\tilde{\nu }}_{3}$) data through Fourier transformation along the $t_{1}$ axis after applying relevant Fourier filters. The resolution along the ${\tilde{\nu }}_{1}$ and ${\tilde{\nu }}_{3}$ axes is 67 and 50 cm^−1^, respectively. Pulse fluences of 1.1 μJ.cm^−2^ (corresponding to an energy per pulse of 0.5 nJ) were used for beams 1 to 3, while the local oscillator was attenuated by a factor of ~100 with the help of a neutral density filter. Absolute phase of the 2DES signal was determined by comparing the appropriate projections to pump–probe spectra measured in the same experimental conditions, using beam 2 as a pump and beam 4 as a probe. About 90 population time values were recorded, ranging from 0 to 500 ps with a variable step increasing exponentially from 20 fs to 50 ps.

Cells of wild type *R. sphaeroides* strain NCIB8253 were grown in M22+ medium (61) in filled 500 mL flat-sided bottles. These were immersed in a glass-sided water bath at 34 °C and illuminated with four 100 W tungsten incandescent light bulbs. Cells were harvested by centrifugation at 18,000 rpm for 20 min and the pellet mixed to homogeneity with an equal volume of glycerol to form a concentrated cell suspension. For measurements, concentrated cells were diluted with a 10-fold volume of 1:2 glycerol:buffer mixture (phosphate-HCl buffer, pH 6.8) in order to obtain an optical density of 0.38 at 850 nm in a fused silica cuvette with an optical path of 0.2 mm. During the measurements, the sample was submitted to a raster scanning motion in the plane perpendicular to the excitation beams by means of a motorized XY translation stage. Motion speed was ~1 to 2 mm/s and different for two directions. Movements on both axes were independent, leading to a zigzag motion covering an area of approximately 4 mm^2^. For a beam size (full width at half maximum) of 200 μm, a given bacterial cell is estimated to remain within the excitation region for approximately 0.1 s. The raster scanning procedure assists in avoiding excess photobleaching of the bacteria and substantially suppresses scattering artifacts, the latter resulting from the destructive interference of rapidly changing coherent scattering. Because of the low excitation intensities and the high scattering contribution, a series of five 2DES measurements was performed on the same sample (60 h overall measurement time) and averaged.

## Supplementary Material

Appendix 01 (PDF)

## Data Availability

Spectroscopic data from Figs. 1–3 have been deposited in a Zenodo repository (62).
