# Supplementary material for: Charting light harvesting in purple bacteria in vivo
Source: Proc Natl Acad Sci U S A. 2026 Jun 29;123(27):e2537487123. doi: 10.1073/pnas.2537487123 (PMC13342825; doi:10.1073/pnas.2537487123)
Supplement: Supplementary file 1 — Appendix 01 (PDF) [file pnas.2537487123.sapp.pdf]

PNAS

Supporting Information for
Charting Light Harvesting in Purple Bacteria *In Vivo*

Romain Rouxel,† Julian Lüttig,†,⊥ Michael R. Jones,‡ and Donatas Zigmantas*,†

† Division of Chemical Physics, Department of Chemistry, Lund University, P. O. Box 124, SE-221 00 Lund, Sweden
⊥ Department of Physics, University of Ottawa, 150 Louis-Pasteur Private, Ottawa, ON K1N 6N5, Canada
‡ School of Biochemistry, Biomedical Sciences Building, University of Bristol, University Walk, Bristol, BS8 1TD, United Kingdom

*To whom correspondence should be addressed.
Email: donatas.zigmantas@chemphys.lu.se

This PDF file includes:

B800 band photobleaching
- Figure S1

Calculation of pigment concentrations and LH2/LH1 ratio
- Figure S2

Calculation of the excitation probability per complex

Probability of double excitation
- Table S1

Ratio of open to closed RCs and annihilation level

Influence of exciton-exciton annihilation on the measured dynamics
- Figures S3 and S4

Additional 2DES measurement at higher pulse energy (2 nJ per pulse)
- Figure S5

Additional 2DES measurement at 80 K
- Figures S6, S7, and S8

SI References

B800 band photobleaching

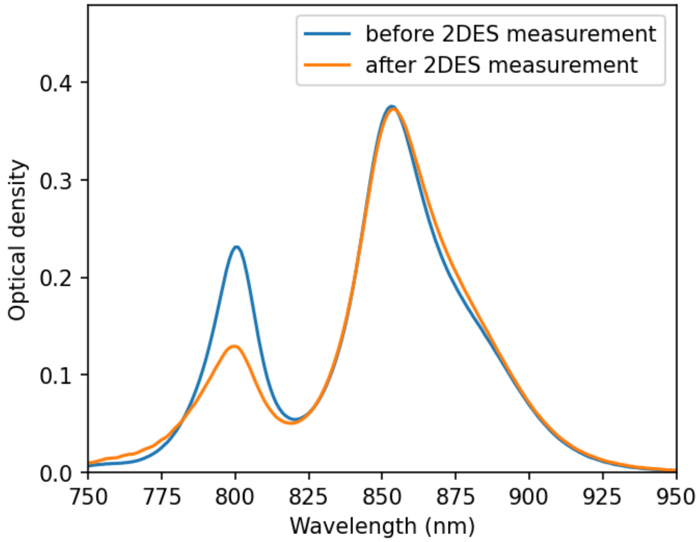

Figure S1. Absorbance spectra of the *R. sphaeroides* sample before and after 2DES measurements at 295 K.

As shown in Figure S1, a ~40 % decay of the B800 band of LH2 is observed between the absorbance spectra measured before (Fig. S1, blue solid) and after the 2DES measurement (Fig. S1, orange solid), while the B850 and B875 bands remain nearly identical. This could indicate a partial structural change of LH2 complexes during the measurement. It is not clear whether this change was caused by high laser intensities or by time evolution while at room temperature in the closed cuvette. In contrast to the B850 ring of LH2 and the B875 ring of LH1, the B800 ring consists of only weakly coupled BChl *a* pigments. Previous studies have demonstrated that the B800 pigments of LH2 can be removed or chemically modified, e.g. oxidized, without significantly affecting the B850 pigments or their corresponding absorption spectrum (1, 2). Thus, we assume that by the interaction with ambient or laser excitation light the selective bleaching of the B800 took place in our sample. In each of the five 2DES spectra (averaged to provide the final data), acquired over a total period of ~60 hours, this effect resulted in a slow gradual decay of the peak associated with initial excitation of the B800 band, while the amplitude of peaks linked to B850 or B875 excitation remained identical through the measurement. This gradual photobleaching of B800 should induce an additional slow component in the decay dynamics in the B800 excitation region of the 2DES data. However, there are three distinct reasons why photobleaching has little impact on our global analysis:

- From comparison of the $t_2 = 40$ fs signal amplitudes for each of the five 2DES data sets, the B800 band decay is on average 7% from one acquisition to the other. The amplitude of the additional decay component is thus reasonably low, even though the whole experiment results in noticeable photobleaching.
- In the final 2DES data set, the amplitudes of the signals related to the B800 excitation are low as compared to signals resulting from B850 and LH1 excitation. While this is partially a consequence from the photobleaching of B800, it also implies that these signals with slightly altered dynamics contribute less to the global analysis.
- To verify the former point, global analysis was performed in a spectral region excluding B800 excitation. It led to very similar time constants as the global analysis on the full spectrum, except, as expected, for the absence of the 700 fs component corresponding to B800 → B850 energy transfer.

Having verified the accuracy of the time constants, we chose to present in the main article only the global analysis on the full spectrum, containing the full picture of energy transfer.

Calculation of pigment concentrations and LH2/LH1 ratio

To obtain insight into the proportions of the different complexes, we calculate the BChl concentrations associated with each complex, $c_{BChl,i}$, using the molar extinction spectra found in the literature (3). From Beer's law, the absorbance can be expressed as:

$$A(\lambda) = \sum \varepsilon_i(\lambda) * l * c_{BChl,i}$$

where l is the cuvette length and the ε_i are the molar extinction coefficients related to BChl molecules from (sub)complex i (corresponding to the B800 or B850 ring of LH2, LH1, or RC).

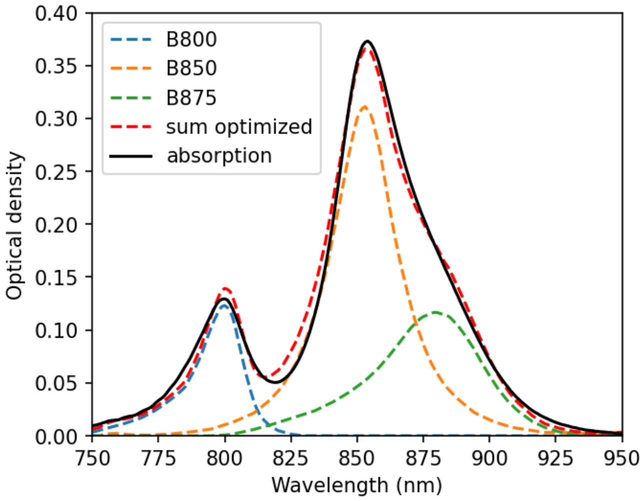

Figure S2. Sample absorbance spectrum (black solid) and fit by a linear combination of the molar extinction coefficient spectra of the different light-harvesting antenna rings types, taken from ref. (3) (red dashed). The individual contributions from the B800 (blue dashed) and B850 (orange dashed) bands of LH2 and the B875 band of LH1 (green dashed) are also shown.

Figure S2 presents the fit of the sample baseline-corrected absorbance spectrum by a linear combination of the molar extinction coefficients from the three types of bacteriochlorophyll (BChl) rings in the light-harvesting antenna complexes. Contribution from the reaction centers (RCs) is omitted as the number of RC pigments is low compared to LH1 and LH2 and its inclusion results in unreliable fits. The factors from the fit correspond to $l * c_{BChl,i}$, with $l = 0.2$ mm, thus allowing us to retrieve the concentrations of BChl belonging to complex i, $c_{BChl,i}$. Further division by the number of BChl per subcomplex (9 for B800, 18 for B850, 56 for the S-shaped LH1) provides the molar concentrations for the complexes, reaching 5.2 µM for LH2 (average of the B800 and B850 concentrations, similar within 1%) and 0.82 µM for LH1. The RC concentration can be considered equal to twice the LH1 concentration. As a main result, we deduce that the sample contains 3.15 LH2 complexes per RC (or 6.3 LH2 per dimeric LH1-RC). This ratio is high for a sample grown under normal light conditions. In the literature, the LH2/RC ratio is reported to vary from ~1 in normal light to ~3 in dark semi-aerobic conditions (4, 5). We note however the high uncertainty of the fit of the absorbance spectrum presented in figure S2 due to the fact that the LH1 B875 band only appears as a weak shoulder. An error in the fit could lead to an overestimation of the LH2/RC ratio in our system.

Calculation of the excitation probability per complex

First, the molar extinction coefficients $\varepsilon_i(\lambda)$ from ref. (3) (expressed in $mM^{-1}\,cm^{-1}$) are converted into absorption cross-sections $\sigma_i(\lambda)$ (in cm^2), through the formula:

$$\sigma_i(\lambda) = \frac{\ln(10) \cdot 10^6 \cdot \varepsilon_i(\lambda)}{N_A}.$$

Then, the laser spectrum from Figure 1 is converted into a spectrum of the density of photons on the sample for the sum of the two excitation pulses (from beams 1 and 2), $\phi(\lambda)$, expressed in photons/cm^2/nm.

Multiplication of $\sigma_i(\lambda)$ and $\phi(\lambda)$ followed by integration over λ provides the probability of absorption of a photon by a BChl molecule from complex *i*. Multiplying this by the number of BChl in the complex reaches the excitation probability of a complex: 2.49 % for LH2, 5.09 % for the S-shaped LH1, and 0.35 % for the RC (with 4 BChl per RC). Note that our analysis provides only an estimate of the excitation density, as several sources of uncertainty prevent an exact evaluation. However, global analysis provides direct access to the magnitude of annihilation, allowing us to a large extend to distinguish exciton-exciton annihilation from other processes such as exciton transfer.

Assuming proportions of 3.15 LH2 and 0.5 LH1 per RC, as calculated above, the total excitation probability of a RC after absorption of two pulses and complete energy transfer, assuming a quantum efficiency of 0.95 (6) (valid in annihilation-free conditions), is thus:
$(3.15 \times 0.0249 + 0.5 \times 0.0509 + 0.0035) \times 0.95 \approx 10\%$.

Our analysis does not provide a detailed picture of the annihilation process itself, for example, if exciton-exciton annihilation occurs predominantly on specific sites. Recently several studies using higher-order spectroscopy investigated the annihilation process in detail showing that annihilation can be used to infer, for example, exciton diffusion in the C_2S_2 supercomplex or the probability that excitons annihilate if they encounter in squaraine polymers (7, 8). However, such investigations are beyond the scope of the current work, but will be of interest in future experiments.

Probability of double excitation

The probability of exciting k times a complex is given by a Poisson distribution:

$$P(k, \mu) = \frac{\mu^k e^{-\mu}}{k!}$$

with μ the expected value of the number of the excitation per complex, calculated above. The results for some values of k are shown in Table S1, showing that events of double excitation of antenna complexes are rare and thus not responsible for the observed annihilation.

complex	$P(k = 0, \mu)$	$P(k = 1, \mu)$	$P(k = 2, \mu)$
LH2 (μ=0.0249)	0.97	2.4E-2	3.0E-4
LH1 (μ=0.0509)	0.95	4.8E-2	1.2E-3

Table S1. Probability of k excitations generated by two pump pulses in LH1 and LH2.

Ratio of open to closed RCs and annihilation level

The above estimations are a starting point to evaluate the fraction of closed RCs and the level of exciton-exciton annihilation in our system, but further calculations necessitate consequent modeling and approximation. A different approach would be to compare our results with previous studies focusing on the effects of the excitation level. Several works have attempted to quantify the fraction of closed RCs in intact *R. sphaeroides* as a function of light intensity, as well as the rate of exciton-exciton annihilation (4, 9, 10). However, comparison between these studies and to our results is not straightforward because of the distinctive excitation wavelengths and repetition rates used. Here, an important precision must be made. Because the RC recombination time (~1 ms) is usually much longer than the interval between pulses (in spectroscopic techniques involving pulsed light), the primary factor influencing the fraction of closed RCs is the light intensity rather than the pulse fluence. On the other hand, to account for exciton-exciton annihilation, the only factor to consider is the pulse fluence, since annihilation events (at least, singlet-singlet annihilation) occur on a timescale much shorter than inverse repetition rate. Consequently, the fraction of closed RCs and the level of exciton-exciton annihilation follow distinct intensity dependencies in experiments with different repetition rates.

Additionally, information about the intensity (or fluence) is not sufficient, but must be converted into the number of excitons formed in the system per unit time (or per pulse). As shown above, this conversion requires knowledge of the extinction coefficients spectra of the various complexes and their amounts in the bacteria.

We thus encourage spectroscopists studying photosynthetic systems to reason in terms of the number of absorbed photons (per pulse or per unit time) rather than intensity. Comparison, in these terms, of the few available intensity-dependent spectroscopic studies on *R. sphaeroides* is out of the scope of the present work, but would be very helpful in order to disentangle signatures of light-harvesting energy transfer processes from the annihilation effects present in many experimental measurements.

Influence of exciton-exciton annihilation on the measured dynamics

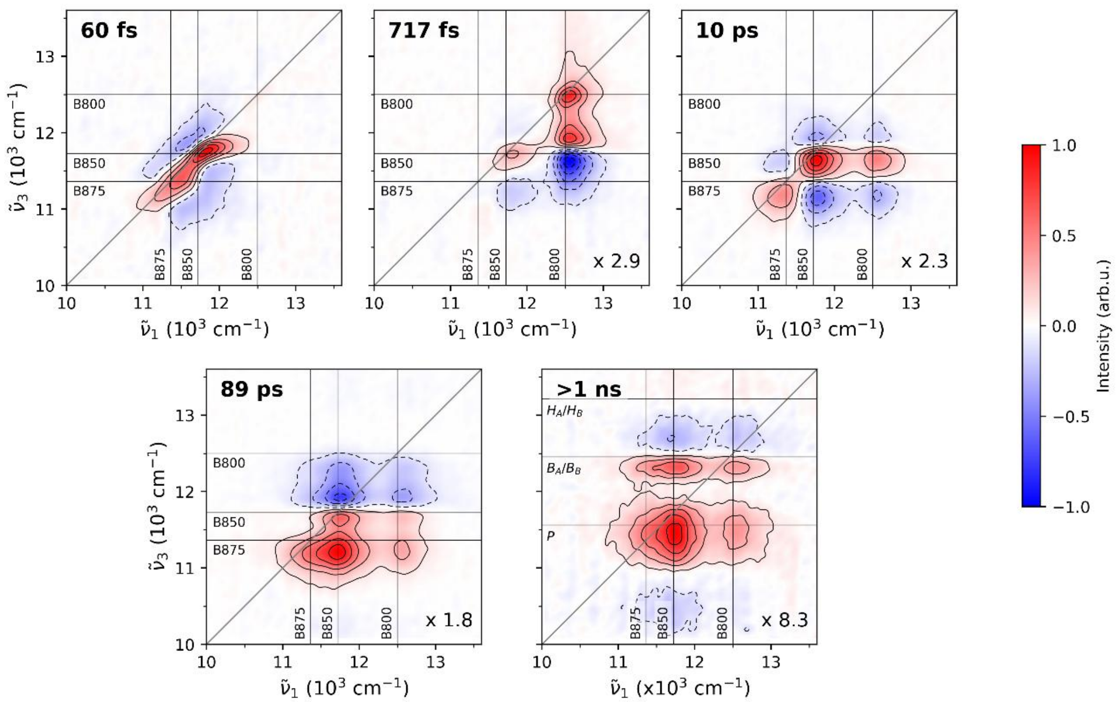

Figure S3. DAS from a global analysis using only five components (legend: see Figure 3 in the main text).

To demonstrate the influence of exciton-exciton annihilation effects on the observed dynamics and the importance of taking them into account during data analysis, we present in Figure S3 results from global analysis of the experimental data using only five components instead of six. While the 60 fs, 700 fs and >1 ns DAS from the 6-component analysis (Figure 3 in the main text) are preserved without notable modification in the 5-component fit, the 7.8 ps, 33 ps and 113 ps DAS are replaced with two 10 ps and 89 ps DAS with mixed spectral signatures. The original 33 ps corresponding to pure exciton-exciton annihilation is absent, and the signatures of this process are visibly present in the two closest components, that were attributed to LH2-LH1 equilibration and LH1-RC transfer.

To get a better estimate of how the annihilation component mixes into various the DAS obtained from the five-component fit, we show spectral cuts along the $\tilde{\nu}_1$ and $\tilde{\nu}_3$ axes (Figure S4). The signal is integrated at the selected $\tilde{\nu}_1$ or $\tilde{\nu}_3$ wavenumbers by summing the signal within ± 100 cm^{-1} around this value to improve the signal-to-noise ratio, resulting in a trace along the corresponding axis. The traces are then normalized by dividing each trace by the maximum absolute signal.

The DAS reflecting exciton-exciton annihilation (Figure 3) exhibits one major positive peak slightly below the diagonal at $\tilde{\nu}_1 = 11400$ cm^{-1} and $\tilde{\nu}_3 = 11200$ cm^{-1}, which we refer to in the following as "peak A". Another positive peak is located at $\tilde{\nu}_1 = 11800$ cm^{-1} and $\tilde{\nu}_3 = 11600$ cm^{-1} ("peak B"), while a weaker negative peak ("peak C") appears at $\tilde{\nu}_1 = 11800$ cm^{-1} and $\tilde{\nu}_3 = 12000$ cm^{-1}. A weak positive and negative feature is observed at the same detection wavelengths but shifted to $\tilde{\nu}_1 = 12500$ cm^{-1}.

In the DAS obtained using five exponentials, the 33 ps component clearly mixes into the 7.8 ps and 113 ps components. Consequently, we expect signatures of peaks A–C at traces for $\tilde{\nu}_1 = 11800$ cm^{-1} and $\tilde{\nu}_3 = 11200$ cm^{-1} in the DAS components with time constants of 10 ps and 89 ps.

We first focus on the comparison between the 7.8 ps and 10 ps DAS components. Figure S4A shows traces at $\tilde{\nu}_1 = 11800$ cm^{-1} for the 10 ps DAS (purple solid line) and the 7.8 ps DAS (green dashed line). At this excitation wavenumber, the 7.8 ps DAS exhibits a negative signal at low detection wavenumbers ($\tilde{\nu}_3 = 11200$ cm^{-1}), followed by a positive signal at higher wavenumbers ($\tilde{\nu}_3 = 11600$ cm^{-1}). Peak B of the annihilation component overlaps with these features, damping the negative signal and enhancing the positive signal. This interpretation is consistent with the 10 ps DAS shown in Figure S4A, which clearly exhibits a weaker negative signal at $\tilde{\nu}_3 = 11100$ cm^{-1}. The influence of peak C is not clearly visible, as it has only a weak amplitude in the 33 ps DAS component.

Figure S4B shows traces along $\tilde{\nu}_3 = 11200$ cm^{-1} for the same two DAS components. The 7.8 ps DAS exhibits a stronger positive peak at $\tilde{\nu}_1 = 11200$ cm^{-1} than the 10 ps DAS. At this detection wavenumber, only peak A contributes to the 33 ps DAS component. Consequently, mixing of the 33 ps DAS, corresponding to annihilation, into the 10 ps DAS results in a stronger positive peak at $\tilde{\nu}_1 = 11400$ cm^{-1}, as observed in the 7.8 ps DAS component.

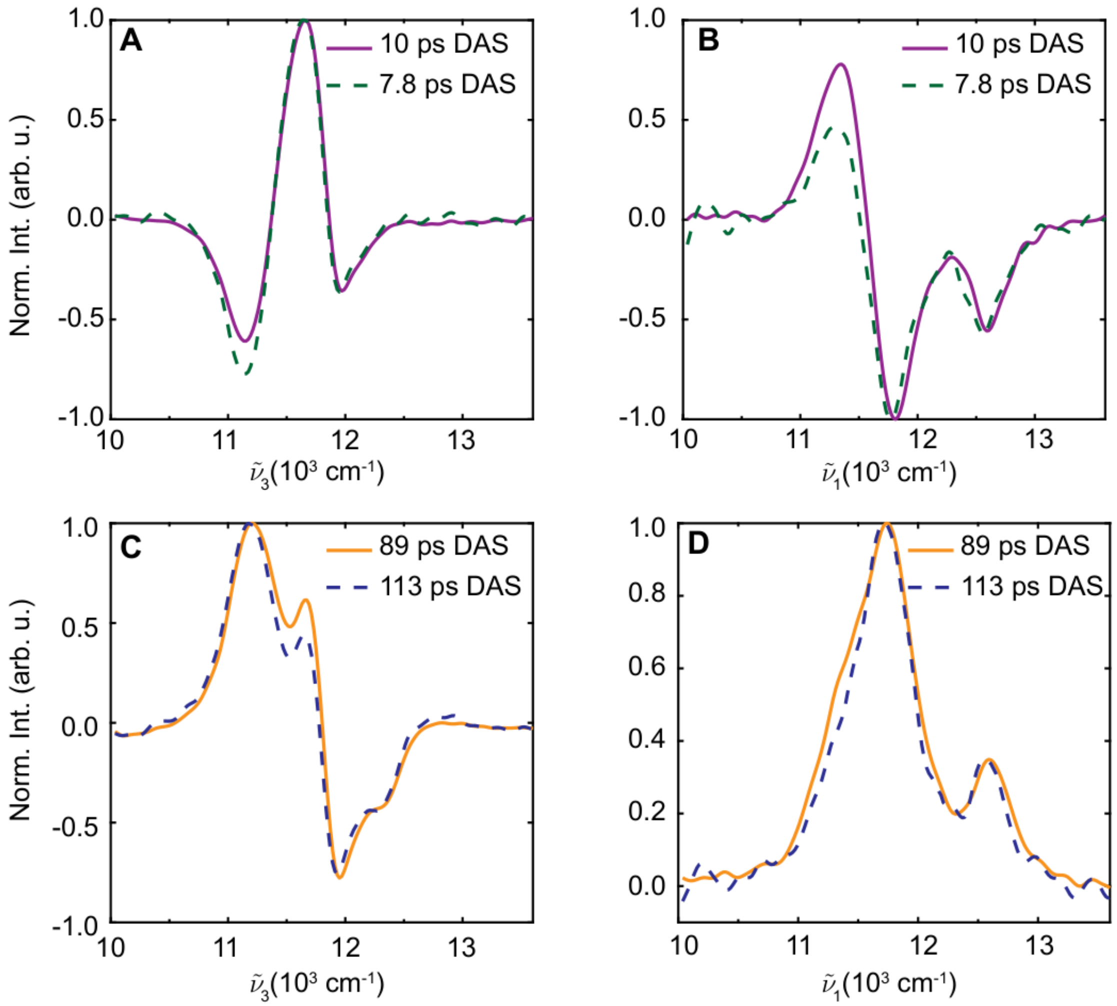

Figure S4. Spectral traces of selected DAS components along $\tilde{\nu}_1$ and $\tilde{\nu}_3$, obtained by integrating over a narrow spectral window, ± 100 cm^{-1} around the central wavenumber. (A) Traces along $\tilde{\nu}_1 = 11800$ cm^{-1} for the 7.8 ps DAS component (green dashed line) obtained from the six-component fit and for the 10 ps DAS component (purple solid line) obtained from the five-component fit. (B) Traces along $\tilde{\nu}_3 = 11200$ cm^{-1} for the 7.8 ps (green dashed line) and 10 ps (purple solid line) DAS

components. (C) Traces along $\tilde{\nu}_1 = 11800$ cm^{-1} for the 113 ps DAS component (blue dashed line) obtained from the six-component fit and for the 89 ps DAS component (yellow solid line) obtained from the five-component fit. (D) Traces along $\tilde{\nu}_3 = 11200$ cm^{-1} for the 113 ps (blue dashed line) and 89 ps (yellow solid line) DAS components.

In Figures S4C and S4D, we compare the DAS components with time constants of 89 ps (yellow solid line) and 113 ps (blue dashed line) at $\tilde{\nu}_1 = 11800$ cm^{-1} and $\tilde{\nu}_3 = 11200$ cm^{-1}, respectively. In Figure S4C, the 89 ps DAS exhibits a stronger positive signal at $\tilde{\nu}_3 = 11700$ cm^{-1} compared to the 113 ps DAS. This behavior can be explained by contributions of peaks B and C of the 33 ps DAS present in the 89 ps DAS component and absent in the 113 ps DAS component.

Figure S4D compares the same DAS components at $\tilde{\nu}_3 = 11200$ cm^{-1}. The influence of the annihilation component on the 89 ps DAS is primarily visible as a weak shoulder at $\tilde{\nu}_1 = 11400$ cm^{-1}, corresponding to peak A of the 33 ps DAS. The contribution of peak A to the 89 ps DAS is weaker than in the 10 ps DAS, indicating that annihilation contributes more strongly to the 10 ps DAS component when five components are used.

Clearly the timescales associated with the two processes of LH2 to LH1 equilibration (7.8 ps component) and the LH1-RC transfer (113 ps component) are modified by mixing with the annihilation dynamics.Therefore, extraction of the annihilation component leads to better estimates for these two constants. This result is particularly remarkable considering the low level of annihilation in the system. Indeed, the annihilation component from Figure 3 has a lower amplitude than the two adjacent ones, and its capture by the six exponential fit required precise adjustment of the initial parameters, otherwise leading to convergence of two components and failing to produce six distinct timescales and spectra.

Additional 2DES measurement at higher pulse energy (2 nJ per pulse)

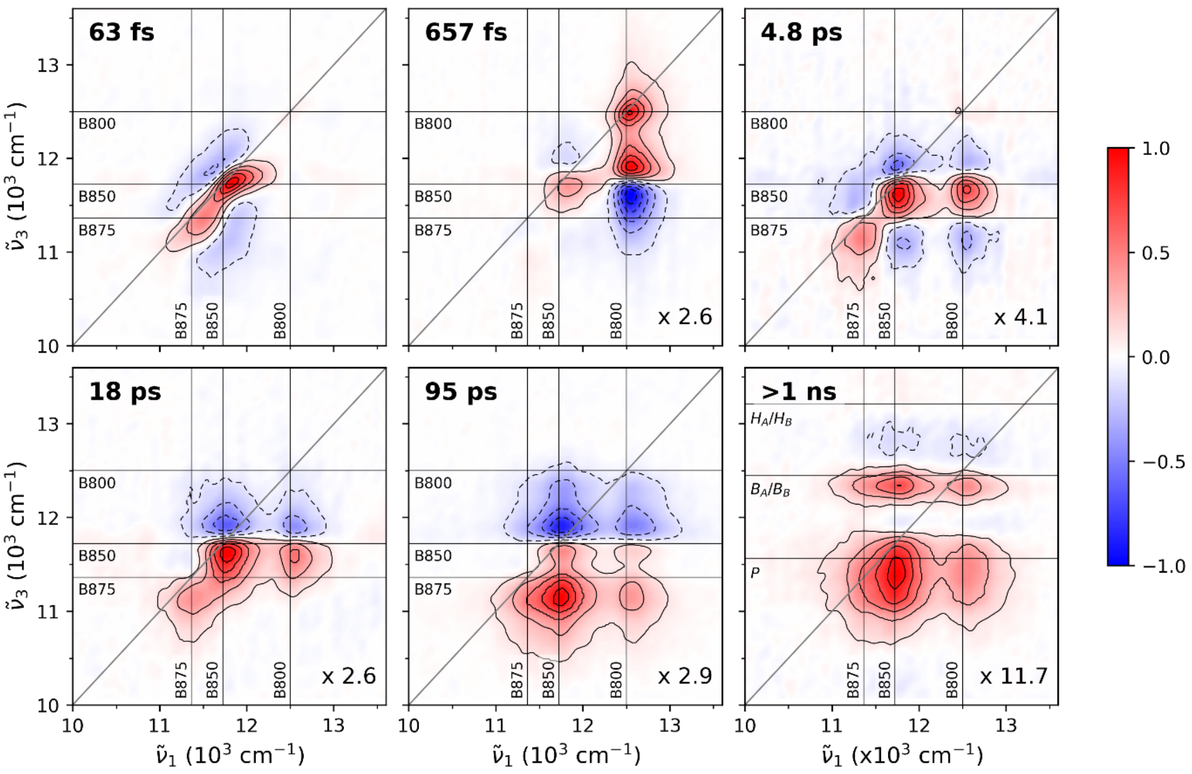

Figure S5. DAS with 6 components from the global analysis of the 2DES data at 296 K and 2 nJ pulse energy (legend: see Figure 3 in the main text).

In order to clarify the identification of the processes observed in the 2DES measurement presented in the main text, performed with a pulse energy of 0.5 nJ for the two pump pulses, we reproduced the same experiment with 2 nJ per pulse. Results from the global analysis of the 2DES data are shown on Figure S5. Our assignment of the six DAS components of Figure S5 is identical to those of Figure 3 in the same respective order. We note a high one-to-one similarity between the DAS of the two experiments, except that the signal-to-noise ratio is considerably higher in the measurement using 2nJ per pulse. Due to the higher signal-to-noise ratio the averaging of different data sets was not required. The differences between the low- and high-power measurements lie in some of the time constants, which are as follows.

- The 4.8 ps component, corresponding to LH2-LH1 energy equilibration, has a shorter timescale than the 7.8 ps component of Figure 3. The most likely explanation is that this component is mixed with exciton-exciton annihilation signal from B850 of LH2 and B875 of LH1, due to similarities in their spectral signatures. In the 4.8 ps DAS of Figure S5, we note that some peaks appear more intense than in the 7.8 ps DAS of Figure 3, as if the latter were superimposed with the annihilation profile of the 18 ps DAS.
- While it is spectrally identical to the 33 ps component of Figure 3, the 18 ps component associated to exciton-exciton annihilation in Figure S5 is 1.8 times faster (18 ps vs. 33ps) and 1.6 times more intense than its counterpart in the 0.5 nJ measurement, after normalization to the amplitude of the 60-66 ps DAS. The faster timescale of the process as well as the nonlinear dependence of the signal on the excitation density are typical signatures of exciton-exciton annihilation. First, with increasing exciton density, the excitons need less time to meet each other and annihilate, resulting in a DAS component

with a faster time constant. Second, the probability of annihilation events increases quadratically with the number of excitons simultaneously present in the antennae network and thus with the energy density. Although the observed amplitude increase with the pulse energy in the DAS component is less than quadratic, this can be explained by the strong leakage of the annihilation dynamics onto other components in the 2 nJ measurement, as observed above in the 4.8 ps DAS. These observations enable us to confirm the assignment of the 33 ps component in the global analysis of the 0.5 nJ per pulse measurement to exciton-exciton annihilation.

- Finally, the 95 ps DAS in Figure S5 is spectrally identical to the 113 ps DAS of Figure 3, meaning that it captures the same processes. This component represents a mixture of the energy transfer from LH1 to open RCs (with timescale ~35-60 ps) and from LH1 to closed RCs (200-250 ps), then one would expect that the time constant for this component should increase along with the proportion of closed RCs. However, despite a four times higher pulse energy, the time constant for this component decreased from 113 ps to 95 ps. An obvious explanation resides once more in the mixing of exciton-exciton annihilation into this component that likely pushes the effective time constant to a shorter value. Compared to the 0.5 nJ measurement, annihilation in LH1 and LH2 is both faster and more intense, especially relative to the other pathways as it causes the loss of excitonic energy that may not reach the RCs.

To conclude, this additional set of measurement strongly supports the assignment of the 33 ps component of the 0.5 nJ measurement to exciton-exciton annihilation in the LH1 and LH2 pools. It also demonstrates the potential influence of annihilation on the timescales associated with other processes and underlines the necessity of the spectroscopic measurements at low excitation power. In our setup, we solve this challenge by a lock-in detection scheme and high stability of the setup allowing to average several data sets and increasing the signal-to-noise-ratio even further. As a side effect of annihilation, power-dependent measurement proved unable to provide answers to the delicate question of the ratio of open to closed RCs. Indeed, the annihilation signal at 2 nJ per pulse “contaminates” the other energy transfer signals and apparently pushes the component associated to the mixed LH1 to RC open and closed towards a shorter timescale in the fit.

Additional 2DES measurement at 80 K

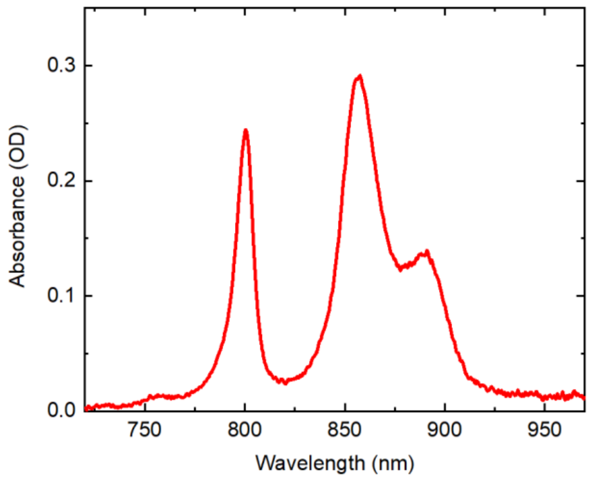

Figure S6. Absorbance spectrum of the *R. sphaeroides* sample before 2DES measurements at 80 K.

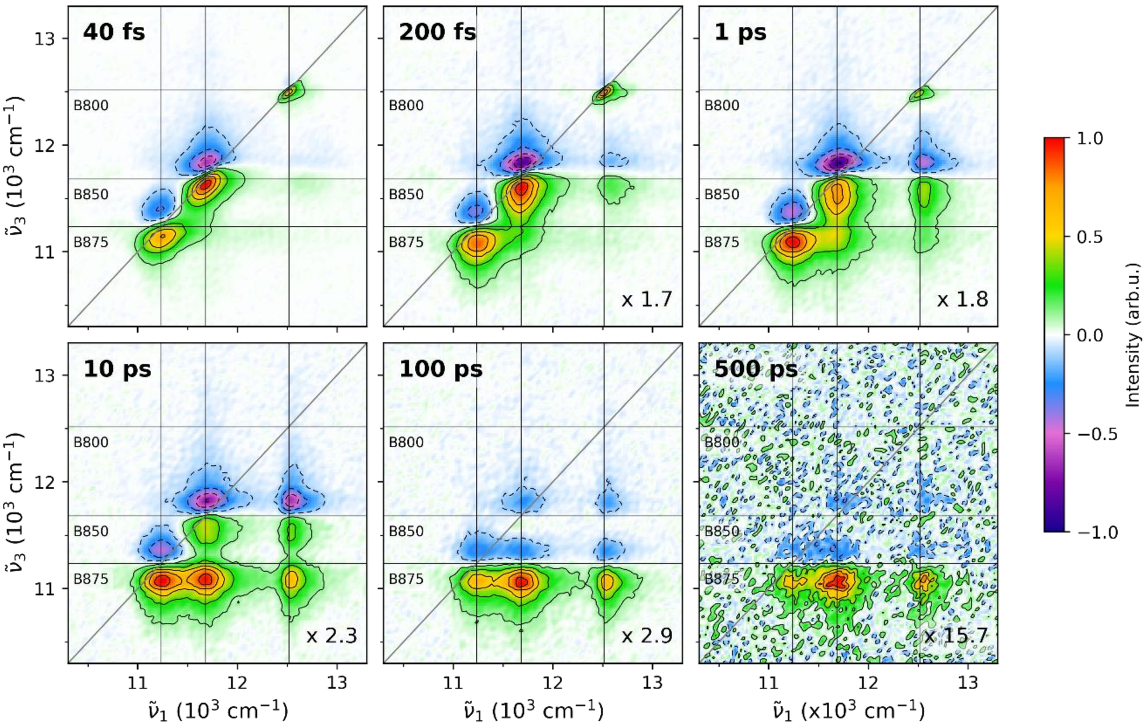

Figure S7. 2DES spectra of *R. sphaeroides* cells at 80 K and 0.5 nJ pulse energy (legend: see Figure 2 in the main text).

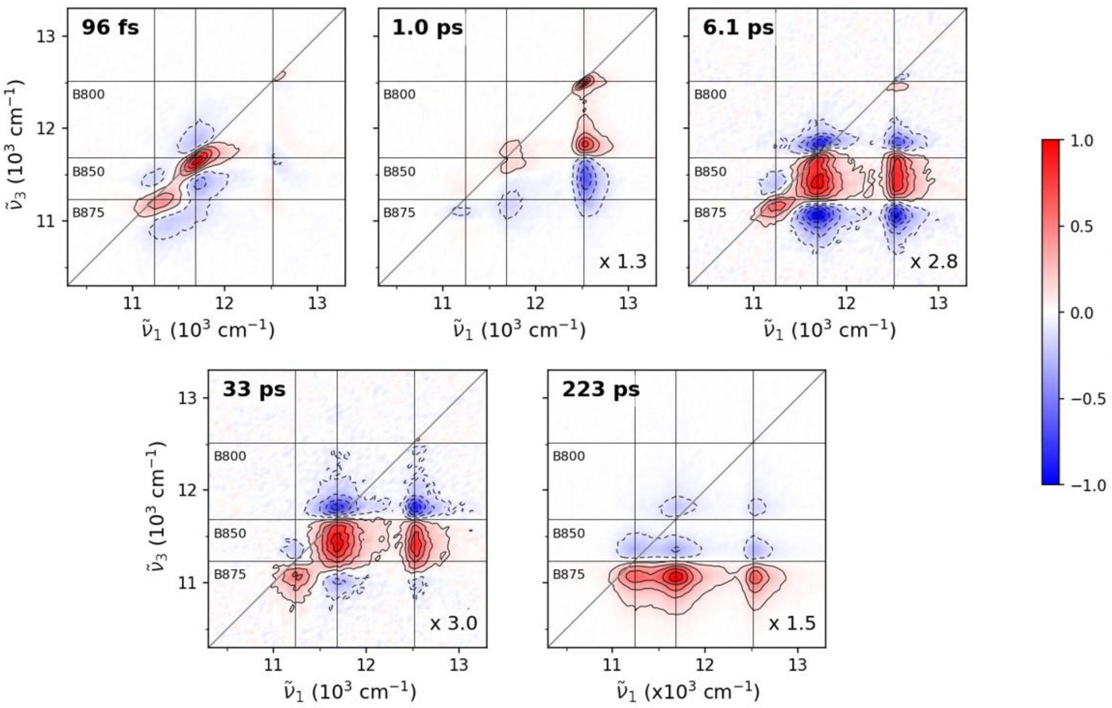

Figure S8. DAS from the global analysis of the 80 K 2DES data with 5 components (legend: see Figure 3 in the main text).

Figures S6, S7 and S8 show the absorbance spectrum, 2DES spectra and DAS, respectively, from a measurement of the *R. sphaeroides* cells at 80 K. Excitation was identical to the room temperature data presented in the main article (1.1 µJ cm^{-2} per pulse). The sample was placed in an optical cryostat (Oxford Optistat DN2) which prevented us from performing XY raster scanning during the measurement. Instead, a single point of the sample was chosen to minimize scattering. The 2DES data (Figure S7) corresponds to the average of six consecutive measurements. No change of the absorbance spectrum was observed during the measurement. Global analysis was performed as described in the main text, resulting in five distinct time constants and DAS (Figure S8).

The first four DAS from the global analysis (Figure S8) are very similar to those obtained at room temperature, with minor modifications of the time constants. The 96 fs time constant corresponds to spectral diffusion in the B875 and B850 rings of LH1 and LH2. The 1.0 ps DAS describes the B800 to B850 energy transfer, in very good agreement with the results of low-temperature measurements in the isolated LH2 complex (11). As in the 700 ps DAS of the room temperature experiment (Figure 3 in the main manuscript), a weak signature of continued B850 spectral relaxation is also observed in the 1.0 ps DAS.

The 6.1 ps DAS mainly corresponds to LH2 to LH1 energy transfer. Its main features are similar to the 7.8 ps component of Figure 3. However, we note a difference in the vertical cut at $\tilde{\nu}_1$ = 11200 cm^{-1} between the measurements at room temperature and 80 K. In Figure S3, the negative feature around (11200 cm^{-1}, 11400 cm^{-1}) has no significant amplitude at $\tilde{\nu}_3$ = 11720 cm^{-1}, energy corresponding to the (GSB, SE) peak of B850. The positive and negative peaks at $\tilde{\nu}_1$ = 11200 cm^{-1} can thus no longer be interpreted as back-transfer from LH1 to LH2, as we did in the room temperature measurement (Figure 3 of the main text). The absence of back-transfer signal is expected, as the process is thermodynamically unlikely at 80 K. The negative peak observed at $\tilde{\nu}_1$ = 11200 cm^{-1} in Figure S3 is in fact ESA signal from LH1 and, along with the positive peak below

it, must be interpreted as a decay of LH1 population, possibly due to early exciton-exciton annihilation between close LH1 neighbors.
The 33 ps component, like its counterpart in the room temperature global analysis (Figure 3), captures the effective dynamics of exciton-exciton annihilation in the LH1 and LH2 pools. Note that the faint negative peaks around (11700 cm^{-1}, 11000 cm^{-1}) and (12500 cm^{-1}, 11000 cm^{-1}) indicate that this process is partially mixed with residual LH2-LH1 transfer that was mainly described by the 6.1 ps component.

As a major difference with the room temperature measurements, no signature of charge separation is observed in the spectra that would indicate energy transfer to open RCs. This can be seen in the 2DES spectra at 100 ps and 500 ps (Figure S7), showing signatures of populated LH1 (and marginally, LH2) states. In the 223 ps DAS (Figure S8), the same spectral shape is observed and corresponds to slow decay of these populations. This decay has been described in previous studies as a combination of fluorescence and quenching by closed RCs. For instance, while fluorescence measurements report a ~1000 ps lifetime in mutant membranes lacking RCs, this lifetime is reduced to ~200 ps when RCs are present but entirely closed. The found value of the LH1 and LH2 population decay, 223 ps, is in agreement with this 200 ps value, which, in addition to the absence of charge separation signal, corroborates the hypothesis that all RCs are closed in the experimental conditions at 80K. The absence of XY scanning of the sample could explain the observed difference between the 80 K and room temperature measurements.

SI References

1. Y. Saga, K. Hirota, H. Asakawa, K. Takao, T. Fukuma, Reversible Changes in the Structural Features of Photosynthetic Light-Harvesting Complex 2 by Removal and Reconstitution of B800 Bacteriochlorophyll *a* Pigments. *Biochemistry* **56**, 3484–3491 (2017).

2. Y. Saga, *et al.*, Selective oxidation of B800 bacteriochlorophyll a in photosynthetic light-harvesting protein LH2. *Sci. Rep.* **9**, 3636 (2019).

3. J. N. Sturgis, C. N. Hunter, R. A. Niederman, Spectra and extinction coefficients of near-infrared absorption bands in membranes of Rhodobacter sphaeroides mutants lacking light-harvesting and reaction center complexes. *Photochem. Photobiol.* **48**, 243–247 (1988).

4. K. Timpmann, *et al.*, Efficiency of light harvesting in a photosynthetic bacterium adapted to different levels of light. *Biochim. Biophys. Acta BBA - Bioenerg.* **1837**, 1835–1846 (2014).

5. P. G. Adams, C. N. Hunter, Adaptation of intracytoplasmic membranes to altered light intensity in Rhodobacter sphaeroides. *Biochim. Biophys. Acta BBA - Bioenerg.* **1817**, 1616–1627 (2012).

6. A. Verméglio, P. Joliot, The photosynthetic apparatus of Rhodobacter sphaeroides. *Trends Microbiol.* **7**, 435–440 (1999).

7. K. Zhang, *et al.*, Probing exciton diffusion dynamics in photosynthetic supercomplexes via exciton–exciton annihilation. *J. Chem. Phys.* **162**, 164201 (2025).

8. P. Malý, *et al.*, Separating single- from multi-particle dynamics in nonlinear spectroscopy. *Nature* **616**, 280–287 (2023).

9. A. Y. Borisov, A. M. Freiberg, V. I. Godik, K. K. Rebane, K. E. Timpmann, Kinetics of picosecond bacteriochlorophyll luminescence in vivo as a function of the reaction center state. *Biochim. Biophys. Acta BBA - Bioenerg.* **807**, 221–229 (1985).

10. M. Onizhuk, S. Sohoni, G. Galli, G. S. Engel, Spatial Patterns of Light-Harvesting Antenna Complex Arrangements Tune the Transfer-to-Trap Efficiency of Excitons in Purple Bacteria. *J. Phys. Chem. Lett.* **12**, 6967–6973 (2021).

11. T. Pullerits, S. Hess, J. L. Herek, V. Sundström, Temperature Dependence of Excitation Transfer in LH2 of *Rhodobacter sphaeroides*. *J. Phys. Chem. B* **101**, 10560–10567 (1997).
